# Emergency liver retransplantation for acute arterial thrombosis in a liver multi-transplanted patient: a case report

**DOI:** 10.1093/jscr/rjaf777

**Published:** 2025-10-03

**Authors:** Priscila Begue Pons, Sol Salgado, Carolina Diego, Juan Pekolj

**Affiliations:** Department of General Surgery, Hospital Italiano de Buenos Aires, Ciudad Autónoma de Buenos Aires C1199ABB, Argentina; Department of General Surgery, Hospital Italiano de Buenos Aires, Ciudad Autónoma de Buenos Aires C1199ABB, Argentina; Department of General Surgery, Hospital Italiano de Buenos Aires, Ciudad Autónoma de Buenos Aires C1199ABB, Argentina; Department of General Surgery, Hospital Italiano de Buenos Aires, Ciudad Autónoma de Buenos Aires C1199ABB, Argentina

**Keywords:** liver retransplantation, hepatic artery thrombosis, vascular reconstruction

## Abstract

Liver retransplantation is a technically demanding procedure, particularly in patients with complex surgical histories and vascular complications such as hepatic artery thrombosis (HAT). We report the case of a 25-year-old woman who underwent a third liver transplant for graft cirrhosis. The procedure was technically demanding due to multiple adhesions, porta hepatic fibrosis, portal hypertension, and hypoplastic vasculature. Arterial reconstruction used an end-to-side anastomosis between the donor’s celiac trunk and the recipient’s splenic artery. On postoperative day 3, she underwent acute HAT and needed an urgent retransplantation (fourth liver). A new arterial anastomosis was created between the origin of the splenic artery and a Carrel patch from the donor’s celiac trunk. The patient made a full recovery. This case highlights the technical challenges of liver retransplantation and the importance of individualized vascular strategies.

## Introduction

Liver transplantation is the definitive treatment for end-stage liver disease. Despite advances that have reduced morbidity and mortality, complications remain common and may necessitate sometimes a retransplantation (RT) [1]. Hepatic artery thrombosis (HAT) is one of the most severe vascular complications, often leading to graft loss [2]. Management options include surgical revascularization, endovascular procedures, or retransplantation.

Retransplantation poses challenges due to the patient’s compromised condition, adhesions, altered anatomy, and complex reconstructions [3, 4].

## Case report

We present the case of a 25-year-old woman with a history of three previous liver transplants—two in childhood for biliary atresia and chronic rejection, and a third in adulthood for graft cirrhosis. Her history included medical and social complexities with irregular follow-up.

The third transplant was performed at our center with a Model for End-stage Liver Disease of 27. The donor was optimal based on clinical and anatomical criteria. Recipient surgery was challenging, due to cirrhotic liver, with massive adhesions and severe portosystemic collateralization. The hepatic artery and portal vein were hypoplastic and unsuitable for anastomosis.

**Figure 1:**
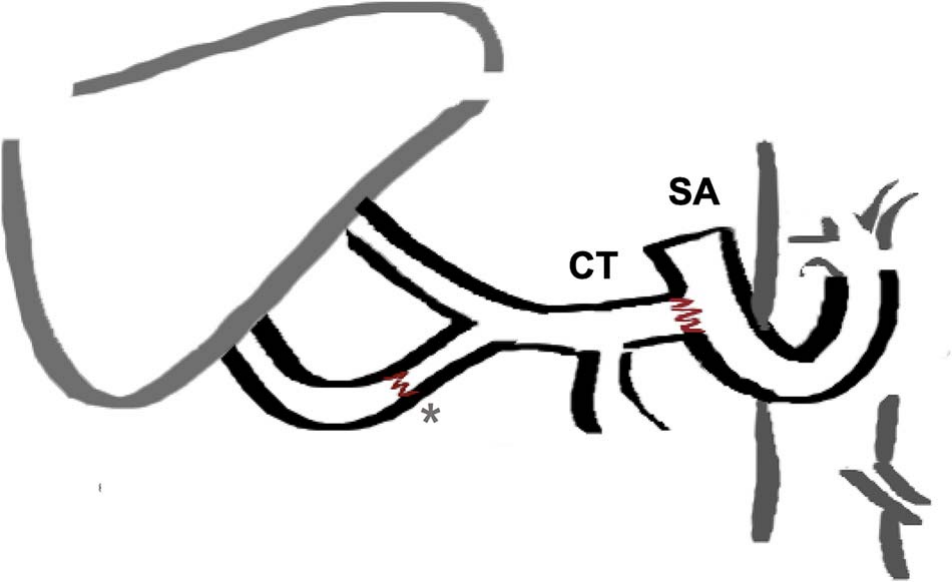
End to side arterial anastomosis between the donor celiac trunk (CT) and the recipient’s splenic artery (SA), which was transected and rotated counterclockwise to the porta hepatis. ^*^Additional anastomosis between gastroduodenal artery and a right hepatic artery originating from the superior mesenteric artery (SMA, donor variant).

After confirming clamp tolerance, total hepatectomy with caval resection was performed. Caval reconstruction was done by end-to-end anastomosis in the proximal and distal segments. Portal inflow was reestablished via a cadaveric venous graft from the mesenteric root to the recipient’s portal vein. The splenic vein and multiple collaterals were ligated to reduce flow steal syndrome. Arterial reconstruction was performed between the donor's celiac trunk and the recipient's splenic artery, which was transected and counterclockwise rotated to porta hepatis area. Due to significant caliber mismatch, an end-to-side anastomosis was performed (Fig. 1).

**Figure 2:**
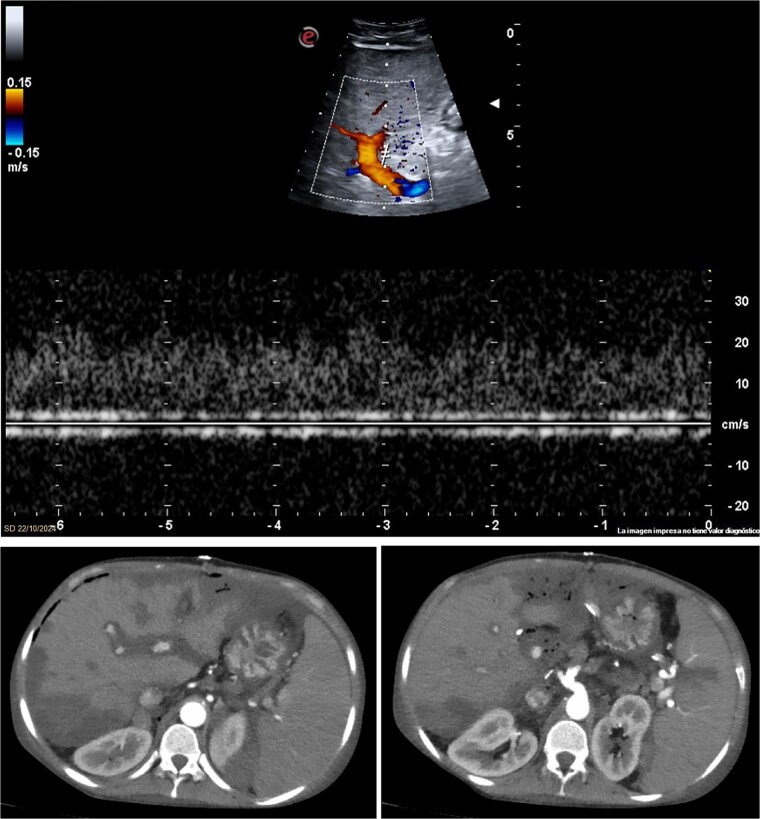
Imaging studies confirming hepatic artery thrombosis. (a) Doppler ultrasound showing absent hepatic artery flow. (b and c) CT angiography showing proximal hepatic artery occlusion with multiple hypodense areas in the liver parenchyma, consistent with ischemia and infarction.

The patient received early antiplatelet therapy due to the high risk of thrombosis. Strict monitoring included lab tests and Doppler ultrasound every 12 h. On postoperative day 3, liver function tests showed no expected improvement, and Doppler failed to detect hepatic artery flow (Fig. 2a). An emergent CT angiography revealed proximal hepatic artery occlusion with multiple hypodense parenchymal areas, consistent with hypoperfusion and infarction (Fig. 2b). In the setting of acute graft failure, the patient was listed for emergency retransplantation. The retrieval process began within 12 h.

**Figure 3:**
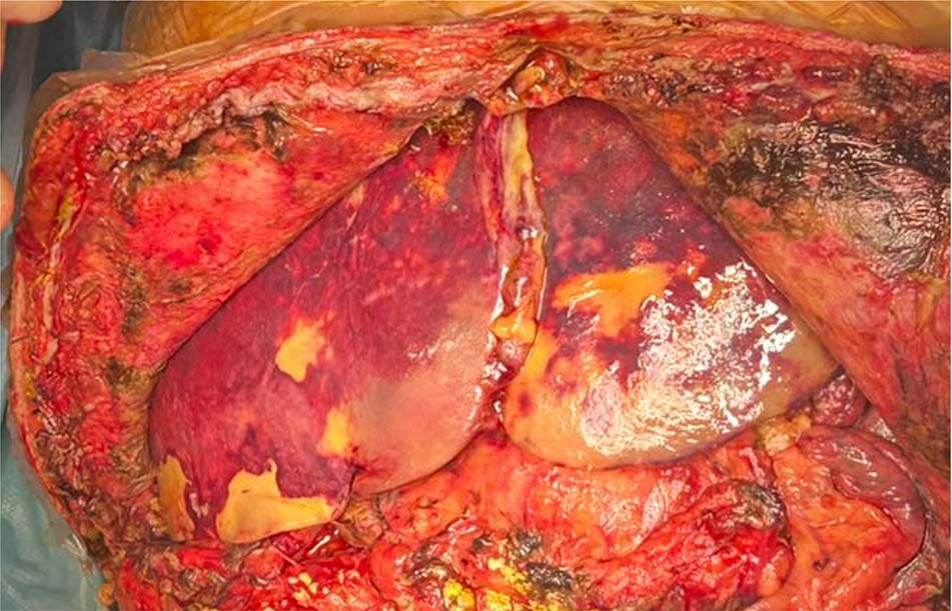
Intraoperative view of enlarged liver with heterogeneous texture and peripheral necrosis.

**Figure 4:**
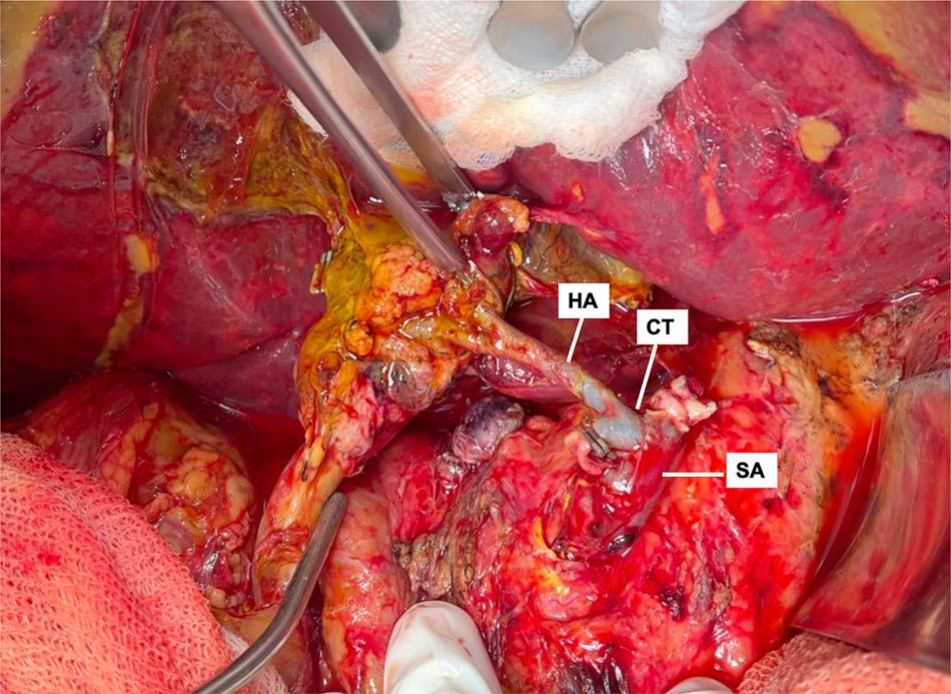
Intraoperative view showing thrombosed hepatic artery (HA) with anastomotic dilation and thrombus extension into the splenic artery (SA).

The liver appeared enlarged, heterogeneous, with peripheral necrosis (Fig. 3). The portal vein and previously constructed mesenteric-portal graft were patent. The hepatic artery was thrombosed throughout its course (Fig. 4). Arterial thrombectomy revealed an extensive clot extending into the splenic artery. Dissection continued proximally until healthy arterial walls and adequate flow were confirmed.

**Figure 5:**
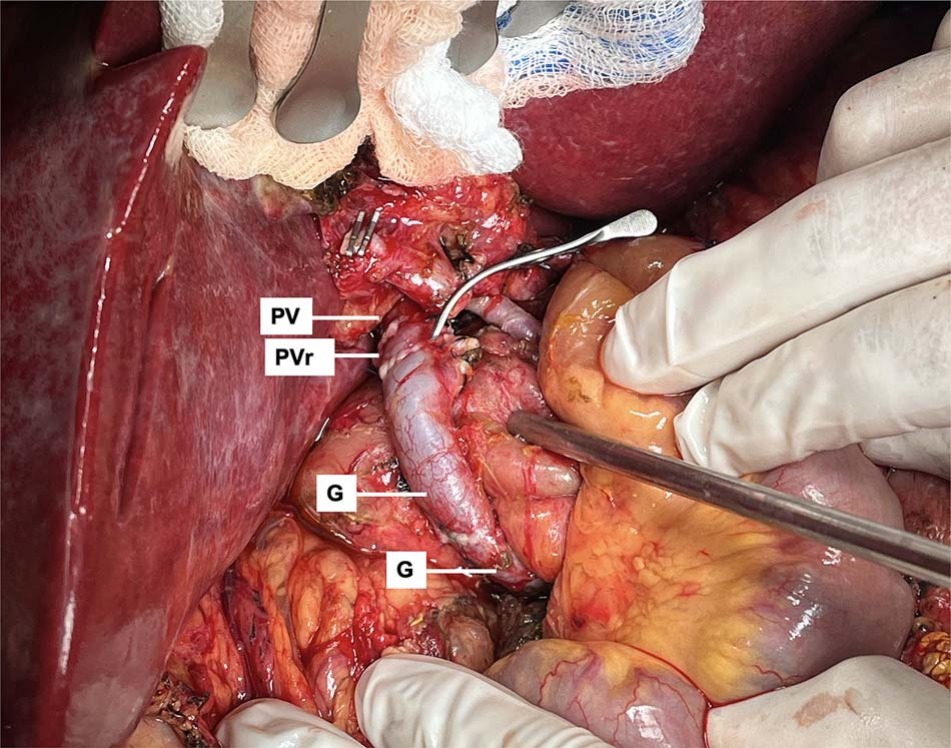
Portal inflow reconstruction using mesenteric to portal shunt with two iliac grafts (G). Anastomosis with portal vein remnant from last transplant (PVr) is shown.

**Figure 6:**
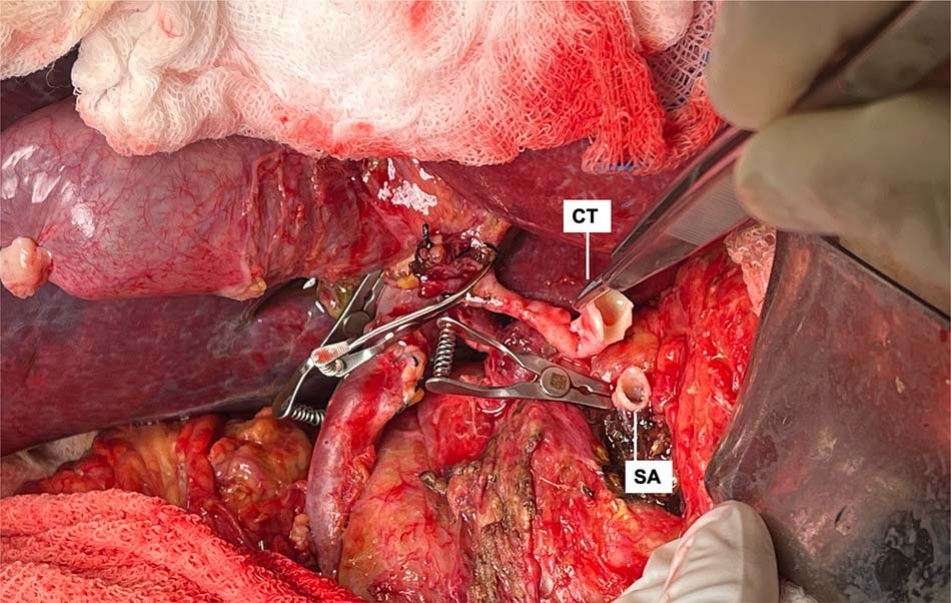
End-to-end arterial anastomosis between the donor celiac trunk (CT) with carrel patch and the recipient’s splenic artery (SA) origin.

**Figure 7:**
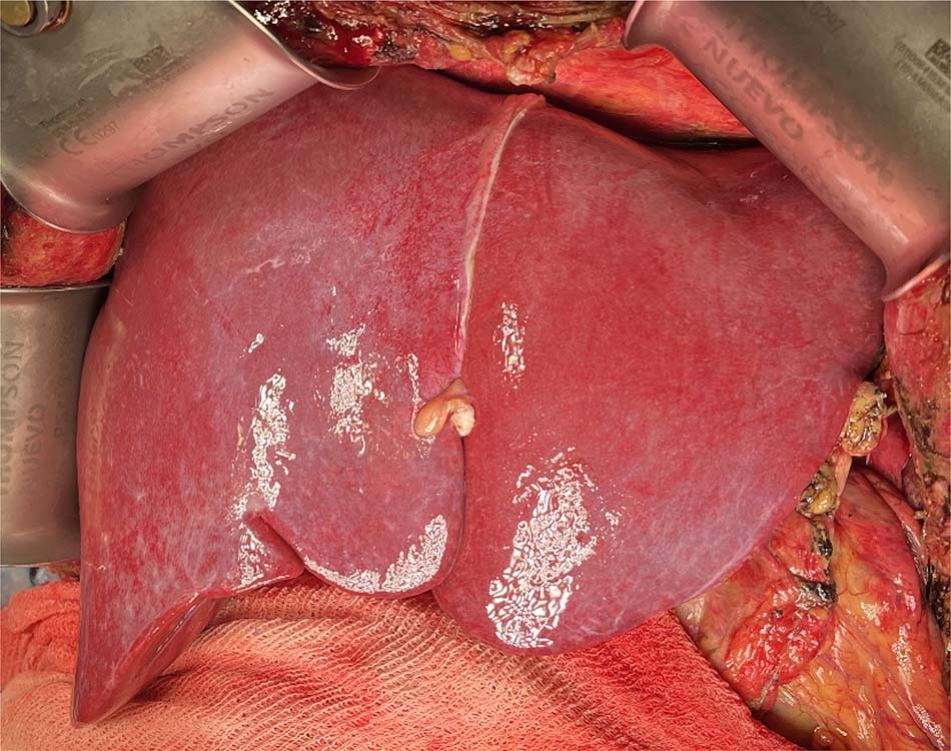
Final view showing homogeneous reperfusion of the liver following vascular reconstruction.

After total clamping and liver explantation, venous reconstruction mirrored the previous technique: cava-to-cava and portal-to-mesenteric graft (Fig. 5). Arterial reconstruction was performed end-to-end between the donor celiac trunk with a Carrel patch and the recipient's splenic artery at its origin (Fig. 6), using two 8-0 polypropylene running sutures. The new liver reperfused uniformly (Fig. 7) and intraoperative Doppler confirmed pulsatile flow. Surgery lasted 3.5 h without complications. The patient had an uneventful ICU and hospital course, with no major postoperative issues and stable outpatient condition at postoperative day 15.

## Discussion

This patient developed early hepatic artery thrombosis (E-HAT), a complication that occurs in 2%–12% of orthotopic liver transplants [1, 2]. Clinical manifestations vary from silent cases to graft failure or death.

Management options include conservative therapy, thrombolysis, urgent revascularization (endovascular or surgical), aorto-hepatic conduit interposition (AHCI), or retransplantation [2, 5, 6]. RT is standard in up to 50% of E-HAT cases, but donor shortages necessitate individualized decisions [2, 3].

In our center, arterial anastomoses are typically performed using the common hepatic artery (CHA) distal to the gastroduodenal artery, which is ligated or used as a confluence patch [3, 6]. In this case, due to CHA narrowing, the splenic artery was selected and anastomosed side-to-end [6–8].

If the splenic artery is unsuitable, an infra-renal AHCI using the donor’s common iliac artery is an alternative [2]. Though demanding, this provides high-pressure flow from a large-caliber source and reduces thrombosis risk [5, 8].

This case highlights the importance of individualized vascular strategies, especially in patients with complex anatomy or multiple prior surgeries. Considering alternative inflow sources helps preserve graft perfusion and reduce the risk of re-thrombosis [1, 4].

## Conclusion

Liver retransplantation remains a challenging procedure. This case illustrates how tailored vascular reconstruction strategies and early recognition of complications are key to graft survival and patient recovery.
